# Effect of Aortic Arch Position on the Bronchial Length Ratio in Situs Determination

**DOI:** 10.1016/j.cjcpc.2026.02.001

**Published:** 2026-02-13

**Authors:** Chae Young Kim, Romina D’Souza, Afsaneh Amirabadi, J.A. Gordon Culham, Shi-Joon Yoo

**Affiliations:** a Department of Diagnostic and Interventional Radiology, Hospital for Sick Children, University of Toronto, Toronto, Ontario, Canada; b Department of Radiology, BC Children’s Hospital, University of British Columbia, Vancouver, British Columbia, Canada; c Division of Cardiology, Labatt Family Heart Centre, Department of Paediatrics, Hospital for Sick Children, University of Toronto, Toronto, Ontario, Canada

**Keywords:** bronchial length ratio, situs, heterotaxy, aortic arch position

## Abstract

**Background:**

Bronchial length ratio (BLR) is widely used for situs determination. However, BLR may also be influenced by the position of the aortic arch. We aimed to evaluate the effect of aortic arch position on BLR.

**Methods:**

Patients were identified using the keywords left aortic arch (LAA), right aortic arch (RAA), double aortic arch, situs inversus, right isomerism, and left isomerism. The ratio between the longer and shorter bronchi was calculated. BLR and bronchopulmonary arterial relationship were compared across groups with different combinations of body situs and aortic arch position.

**Results:**

Patients included 69 with situs solitus and LAA (group 1A), 20 with situs inversus and RAA (group 1B), 43 with situs solitus and RAA (group 2A), 2 with situs inversus and LAA (group 2B), 11 with situs solitus and double aortic arch (group 3), and 14 with right or left isomerism (group 4). Group 2 demonstrated a significantly smaller BLR than group 1 (*P* = 0.001). Group 3 showed a smaller BLR than group 1 but without statistical significance. Group 4 had a significantly smaller BLR than groups 1-3 (*P* < 0.001). None of group 1 had a BLR within the heterotaxy range. BLR ranges of 11% of group 2 and 9% of group 3 overlapped with the heterotaxy range. Bronchopulmonary arterial relationship was consistent with the situs of the remainder of the body in all.

**Conclusions:**

BLR is influenced by aortic arch position, limiting its use for situs determination. Bronchopulmonary arterial relationship should be used as the primary criterion for situs determination.

## Résumé

**Contexte:**

Le rapport des longueurs bronchiques (*bronchial length ratio* ou BLR) est largement utilisé pour déterminer la position des organes dans le corps (situs). Le BLR peut néanmoins être également influencé par la position de la crosse aortique. Nous avons donc cherché à évaluer l’effet de la position de la crosse aortique sur le BLR.

**Méthodologie:**

Des patients ont été recensés à l’aide des mots clés suivants : crosse aortique gauche (CAG), crosse aortique droite (CAD), crosse aortique double, situs inversus, isomérisme droit et isomérisme gauche. Nous avons calculé le rapport entre la bronche la plus longue et la bronche la plus courte. Le BLR ainsi que la relation entre les bronches et les artères pulmonaires ont été comparés entre des groupes présentant différentes combinaisons de situs corporel et de position de la crosse aortique.

**Résultats:**

Nous avons trouvé 69 patients présentant un situs solitus et une CAG (groupe 1A), 20 patients présentant un situs inversus et une CAD (groupe 1B), 43 patients présentant un situs solitus et une CAD (groupe 2A), 2 patients présentant un situs inversus et une CAG (groupe 2B), 11 patients présentant un situs solitus et une crosse aortique double (groupe 3) et 14 patients présentant un isomérisme droit ou gauche (groupe 4). Le BLR était significativement moindre dans le groupe 2 que dans le groupe 1 (*p* = 0,001). Le BLR était aussi moindre dans le groupe 3 que dans le groupe 1, mais cette différence n’était pas statistiquement significative. Le BLR était significativement moindre dans le groupe 4 que dans les groupes 1 à 3 (*p* < 0,001). Aucun des patients du groupe 1 ne présentait un BLR se situant dans la plage correspondant à une hétérotaxie. Les plages de BLR dans les groupes 2 et 3 (11 % et 9 %, respectivement) se si- tuaient dans la plage correspondant à une hétérotaxie. La relation entre les bronches et les artères pulmonaires correspondait au situs dans le reste du corps chez l’ensemble des patients.

**Conclusions:**

Le BLR est influencé par la position de la crosse aortique, ce qui limite son utilisation pour la détermination du situs. Le principal critère utilisé pour déterminer le situs devrait donc être la relation entre les bronches et les artères pulmonaires.

The internal organs in the human body, such as the lungs, bronchi, liver, and spleen, are arranged normally in an asymmetric manner. *Situs solitus* refers to this normal asymmetric arrangement of the internal organs of the body relative to the midline or sagittal plane.^1^^,^^2^ *Situs inversus* is a mirror-image arrangement of these organs. *Heterotaxy* refers to uncommon situations where the situs is neither normal nor mirror-imaged.^1^^,^^2^ In heterotaxy, paired organs–such as the lungs, bronchi, and atrial appendages–usually display a symmetric arrangement, classified as *right isomerism* or *left isomerism* depending on whether both sides resemble the right- or left-sided morphology of situs solitus.^1^^,^^3^, ^4^, ^5^ Determination of body situs is important in patients with congenital heart disease as abnormal situs, particularly heterotaxy, is frequently associated with complex lesions including abnormal systemic and pulmonary venous connections.^1^^,^^2^

**Figure 1:**
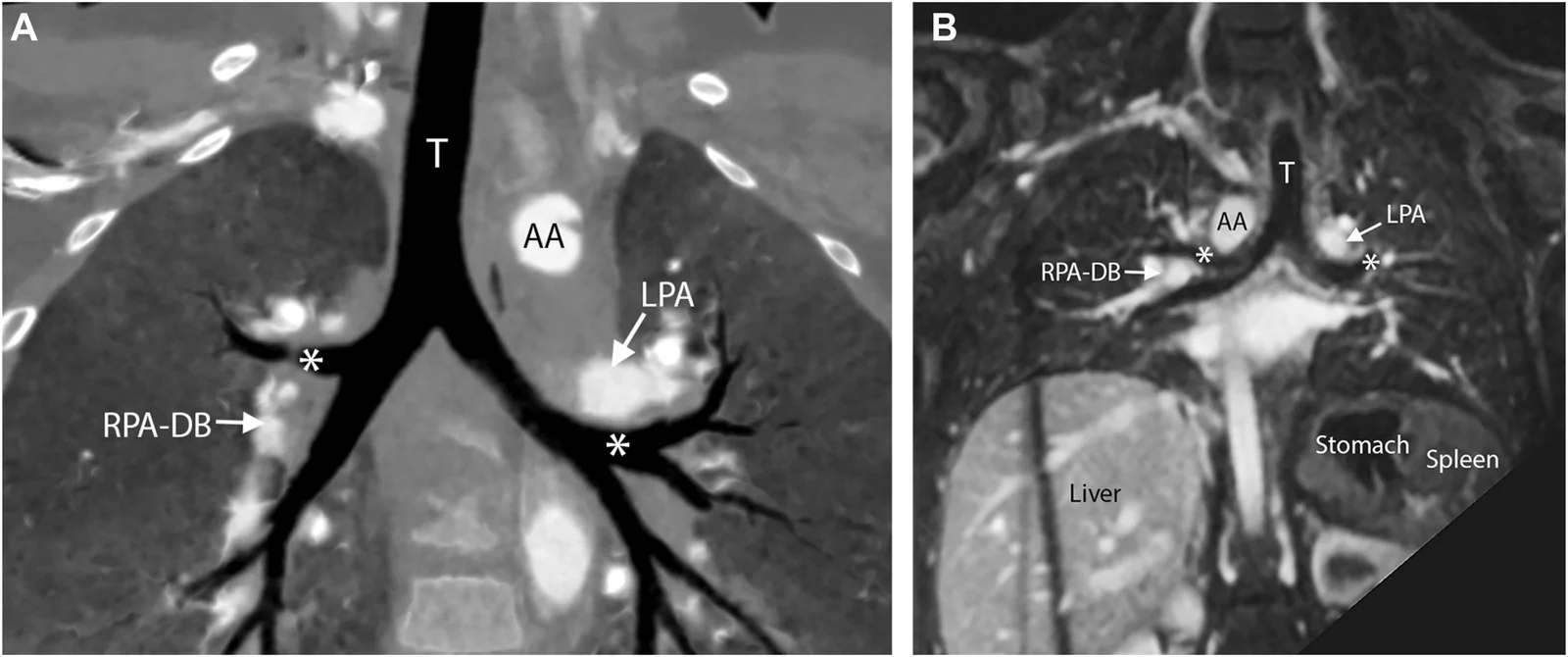
Typical asymmetric bronchial lengths seen in situs solitus with a left aortic arch (**A**) and more symmetric bronchial lengths in a patient with a right aortic arch (**B**). Both cases show a normal relationship between the upper lobar bronchi (**asterisks**) and the pulmonary arterial branches. The left pulmonary artery (LPA) courses backward above the left main bronchus, whereas the descending branch of the right pulmonary artery (RPA-DB) courses backward below the right main bronchus. AA, aortic arch; T, trachea.

The bronchial length ratio (BLR) has long been used as a practical tool for situs determination.^6^, ^7^, ^8^, ^9^, ^10^, ^11^, ^12^, ^13^ However, BLR does not always correspond with atrial situs or the arrangement of other organs.^10^^,^^11^ In clinical practice, we observed several cases of right aortic arch (RAA) in which the BLR falsely suggested an isomeric pattern despite an otherwise normal organ arrangement (Fig. 1). We, therefore, hypothesized that the bronchus on the same side of the aortic arch can be elongated due to the chronic mechanical effect of the latter on the former and that the BLR may not be a reliable indicator of the bronchial situs. The present study aimed to evaluate the impact of aortic arch position on BLR and to assess the reliability of BLR for body situs determination.

## Methods

### Study population

This retrospective study was approved by the institutional research ethics board with a waiver of informed consent (IRB No. 1000080495). The study was performed using deidentified data. Using the radiology database from 2001 to 2022, patients were identified with the keywords left aortic arch (LAA), RAA, double aortic arch (DAA), situs inversus, heterotaxy, and isomerism. Patients with high-quality computed tomographic angiograms (CTA) or magnetic resonance angiograms (MRA) allowing multiplanar reconstruction of the airways were included. Patients with indeterminate or disharmonious situs across the body parts as previously defined^2^ were excluded.

### Data collection and analysis

Demographic data, including sex, age at CTA or MRA, and major cardiovascular diagnosis, were recorded. Patients were categorized into the following groups:

• Group 1. Aortic arch in the usual position for the given situs

Group 1A. Situs solitus with LAA (SS-LAA)

Group 1B. Situs inversus with RAA (SI-RAA)

• Group 2. Aortic arch on the opposite side of the usual position for the given situs

Group 2A. Situs solitus with RAA (SS-RAA)

Group 2B. Situs inversus with LAA (SI-LAA)

• Group 3. Situs solitus with DAA (SS-DAA)
• Group 4. Heterotaxy.

CTA and MRA images were reviewed using AW Server 3.2 (GE HealthCare, Chicago, MN) for 3-dimensional reconstruction.

**Figure 2:**
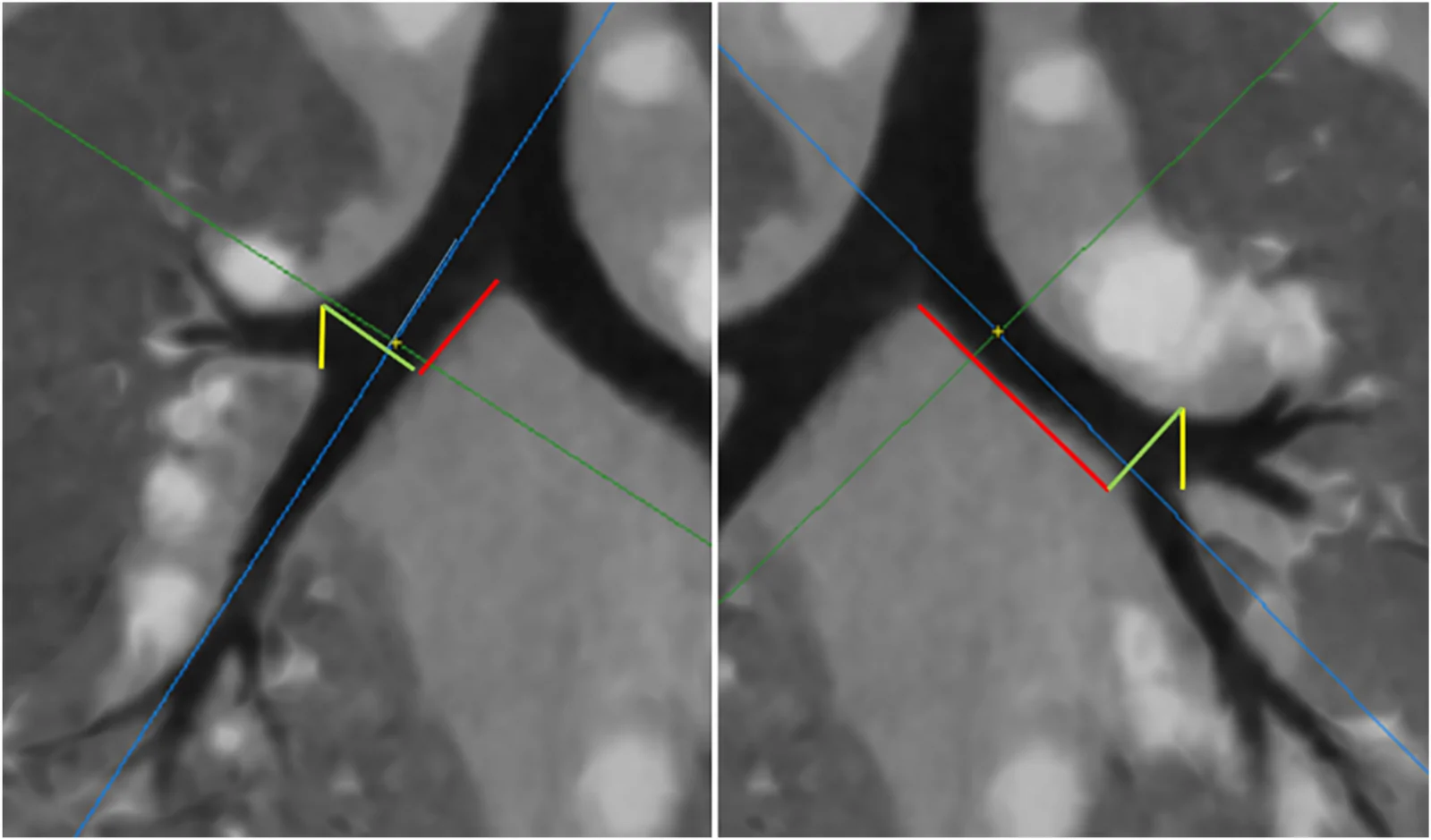
Computed tomograms showing how the bronchial lengths are measured after each bronchus is reconstructed along its long axis. Step 1. Draw a line (**yellow**) across the upper lobar bronchus from the inferior point of origin. Step 2. Draw a line (**green**) across the lower end of the main bronchus from the upper tip of the **yellow line**. Step 3. Measure the length (**red line**) from the inferior tip of the **green line** to the carinal bifurcation point.
• BLR: ratio of the longer to shorter main bronchus
• Aortic arch position relative to the trachea: left, right, or double.

The following variables were assessed:

• Bronchopulmonary arterial relationship:
  ○ Eparterial bronchus (upper lobar bronchus above the pulmonary artery)
  ○ Hyparterial bronchus (upper lobar bronchus below the pulmonary artery)
  ○ Not definable
• Right and left main bronchial lengths (Fig. 2)

### Statistical analysis

Statistical analyses were performed using RStudio (Posit Software, Boston, MA). Continuous variables were non-normally distributed and are reported as mean ± standard deviation, median (interquartile range), and range. Group comparisons of BLR were performed using the Kruskal-Wallis test, with Dunn’s *post hoc* test and Bonferroni correction for multiple comparisons. Two-group comparisons were performed using the Wilcoxon rank-sum (Mann-Whitney) test. Patients were categorized a priori by situs and aortic arch position; the small subgroup of situs inversus with LAA (n = 2) was described but not analyzed inferentially. All tests were 2-sided, and *P* < 0.05 was considered statistically significant.

## Results

**Figure 3:**
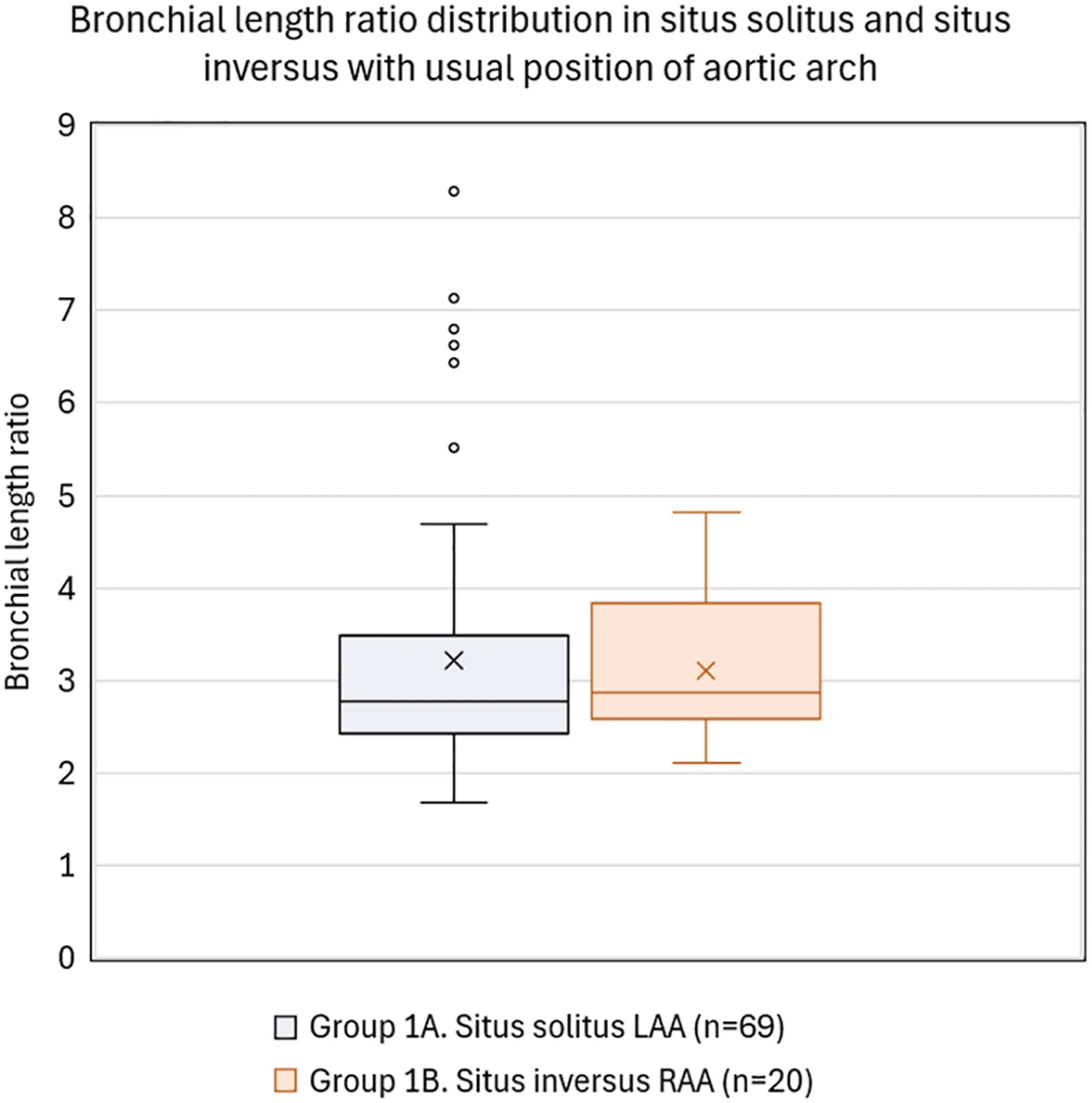
Box-and-whisker plots showing the bronchial length ratio distribution in situs solitus with left aortic arch (LAA) (group 1A) and situs inversus with right aortic arch (group 1B).

**Figure 4:**
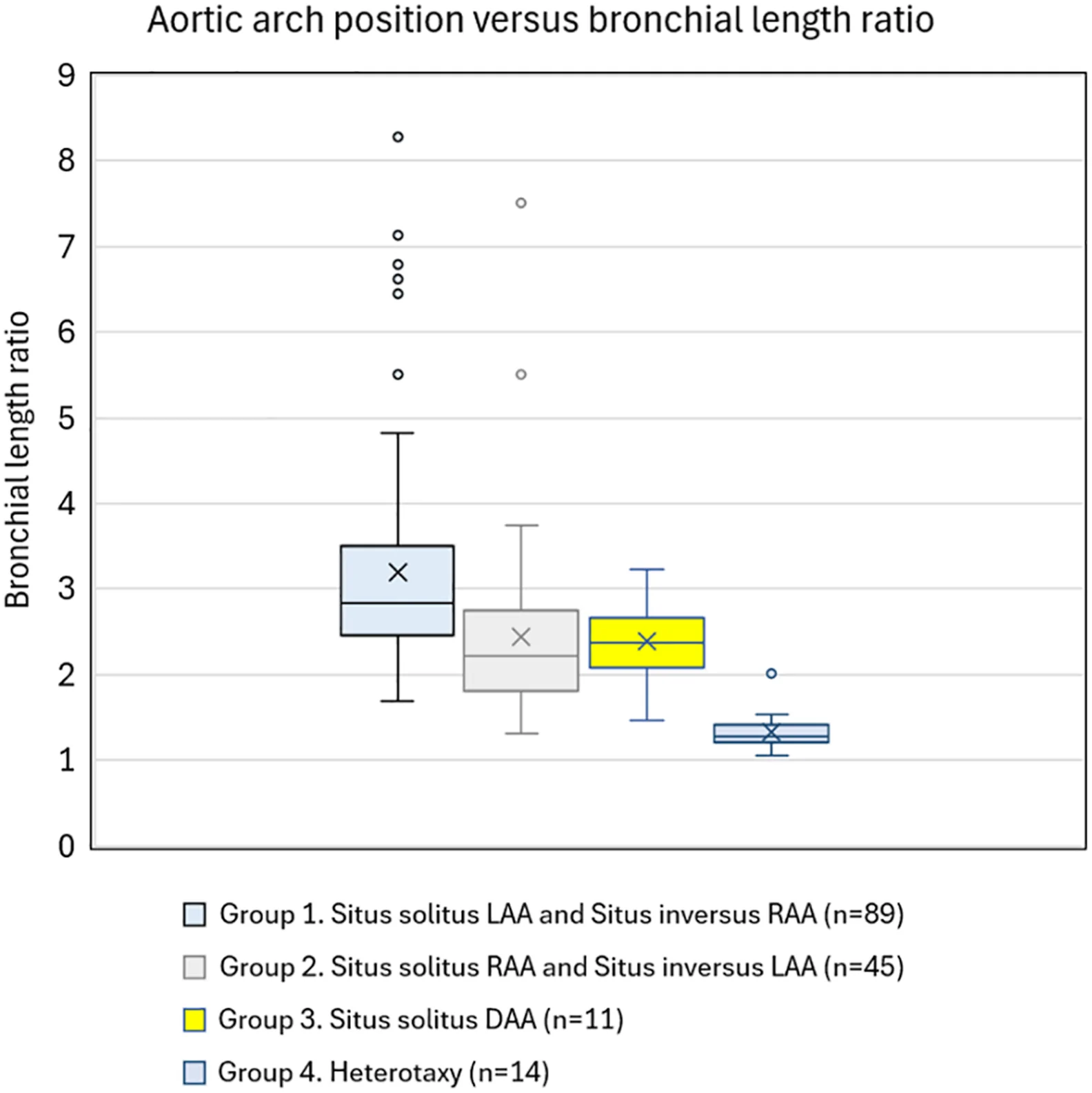
Box-and-whisker plots showing the distribution of the cases in 4 study groups. DAA, double aortic arch; LAA, left aortic arch; RAA, right aortic arch.

The study population included 159 patients (89 male, 70 female). Age distribution was non-normal across all groups (*P* value <0.01). The mean age was 6.7 years (median age 5.2 years; range: 0 day to 17.9 years. Patient characteristics by group are summarized in Table 1. BLR distributions are shown in Tables 2,3,4 and Figures 3 and 4.

**Table 1:** Patient groups and characteristics

	Age	Sex (M:F)	CTA/MRA
Group 1A. Situs solitus with left aortic arch (n = 69)	Mean: 6.22 Median: 3.45 (0 days to 17 years)	38:31	41/28
Group 1B. Situs inversus with right aortic arch (n = 20)	Mean: 8.90 Median: 10.02 (3 days to 17 years)	13:7	11/9
Group 2A. Situs solitus with right aortic arch (n = 43)	Mean: 8.15 Median: 8.14 (1 day to 16 years)	24:19	25/18
Group 2B. Situs inversus with left aortic arch (n = 2)	Mean: 10.54 Median: 10.54 (5 years to 15 years)	1:1	0/2
Group 3. Situs solitus with double aortic arch (n = 11)	Mean: 2.76 Median: 0.50 (100 days to 16 years)	7:4	9/2
Group 4. Heterotaxy (n = 14)	Mean: 4.44 Median: 1.39 (68 days to 16 years)	6:8	5/9

CTA, computed tomographic angiogram; F, female; M, male; MRA, magnetic resonance angiogram.

**Table 2:** Distribution of bronchial length ratio in the study groups

Group	Group 1			Group 2			Group 3	Group 4
	1A + 1B	1A	1B	2A + 2B	2A	2B		
Sample size	89	69	20	45	43	2	11	14
Mean	3.20	3.23	3.11	2.44	2.47	1.64	2.38	1.34
Median	2.84	2.77	2.87	2.22	2.24	1.64	2.38	1.28
Maximum	8.28	8.28	4.82	7.51	7.51	1.96	3.23	2.02
Minimum	1.68	1.68	2.11	1.31	1.35	1.31	1.47	1.06
SD	1.21	1.32	0.75	1.08	1.09	0.46	0.44	0.24

SD, standard deviation.

**Table 3:** Statistical significance of difference between the groups (Dunn’s multiple comparison test)

Comparison groups	Mean rank difference	Significance	Adjusted *P* value
Group 1A vs group 1B	-0.50	Not significant	1.00
Group 1 vs group 2	4.54	Significant	<0.0001
Group 1 vs group 3	2.33	Not significant	0.06
Group 1 vs group 4	6.89	Significant	<0.0001
Group 2 vs group 4	3.75	Significant	<0.001
Group 3 vs group 4	3.07	Significant	0.01

**Table 4:** Statistical significance between group 4 and groups 1-3 (Wilcoxon rank-sum test)

Comparison group	Significance	*P* value
Group 4 vs groups 1-3	Significant	<0.0001

For comparisons between groups, the Kruskal-Wallis test results indicated significant differences between at least 2 of the study groups (*P* < 0.05).

The subgroups in group 1 (patients with aortic arch in the appropriate position for the given situs) showed no significant difference in distribution of the BLR (adjusted *P* value = 1.00) (Table 2 and Fig. 3). There were 6 outliers in group 1A, all of which were above the upper extreme. The combined group of these 2 subgroups (group 1) showed an interquartile range of 2.47-3.49, with a mean BLR of 3.20.

Group 2 (patients with aortic arch on the opposite side of the usual position for the given situs) demonstrated a significantly smaller BLR than group 1 (adjusted *P* value <0.0001) (Tables 2 and 3, and Fig. 4). There were 2 outliers, both of which were above the upper extreme.

Group 3 (situs solitus with DAA) showed a BLR distribution similar to group 2, although the difference compared with group 1 did not reach statistical significance (adjusted *P* = 0.06) (Tables 2 and 3, and Fig. 4).

**Figure 5:**
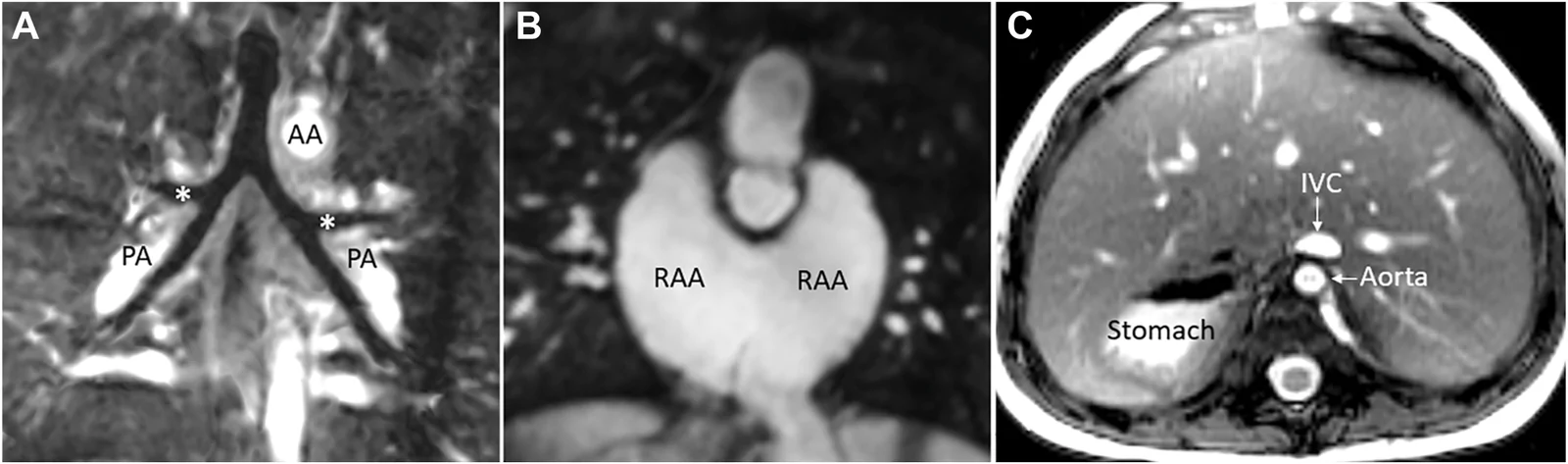
Magnetic resonance images from a patient with heterotaxy, right isomerism of the atrial appendages, and asplenia. (**A**) The bronchial length is asymmetric with a ratio of 2.02. However, the upper lobar bronchus is above the descending branch of the ipsilateral pulmonary artery (PA) on both sides. (**B**) The appendages of both atria are triangular with a wide orifice, which is characteristic of the morphologically right atrial appendage (RAA). (**C**) There is no spleen behind the stomach. The inferior vena cava (IVC) and abdominal aorta are juxtaposed on the left side of the spine. AA, aortic arch.

Group 4 (heterotaxy) showed a narrow distribution with a mean of 1.34 and was significantly different from groups 1-3 (*P* < 0.001) (Tables 2 and 4, and Fig. 4). One outlier (BLR of 2.02) exhibited other classic features of right isomerism, including bilateral eparterial bronchi (Fig. 5). In this group, the bronchus on the same side of the aortic arch was longer than the bronchus on the other side in 89% of cases.

No patient in group 1 had a BLR overlapping the heterotaxy range (group 4). Five patients (11%) in group 2 and 1 patient (9%) in group 3 demonstrated BLR values within the heterotaxy range (group 4).

In all patients, the relationship between the branch pulmonary artery and the ipsilateral main bronchus was in accordance with the situs of the remainder of the body.

## Discussion

Determination of the situs is the first step of the sequential segmental analysis of congenital heart disease. The arrangement of the lungs and bronchi, the arrangement of the atrial appendages, and the splenic status are usually harmonious.^2^^,^^11^, ^12^, ^13^ Although it is often difficult or inaccurate to determine the atrial situs based on the shape of the atrial appendages and distribution of the pectinate muscles, the thoracic situs determination based on the bronchial branching seen in CT or MR is generally straightforward and reliable.^2^

Two bronchial features are commonly used for situs determination: the bronchopulmonary arterial relationship and the BLR. Consistent with prior studies,^2^^,^^10^ our findings confirm that the bronchopulmonary arterial relationship is a more reliable indicator of situs than BLR. However, the BLR has widely been used, and a ratio of 1.5 between the longer and shorter bronchi is a broadly accepted criterion for the differentiation of the lateralized and isomeric situses.^11^ In our study, none of the individuals having situs solitus or inversus with an appropriate position of the aortic arch had a BLR below 1.5. The BLR was equal to or less than 1.5 in all patients with heterotaxy except for one. This outlier had all the classic features of right isomerism, including bilateral eparterial bronchi, but the bronchial lengths were markedly asymmetric (Fig. 5).

In contrast, 11% of patients with an atypical aortic arch position for the given situs and 9% of those with DAA demonstrated BLR values overlapping with heterotaxy. These findings suggest that aortic arch position alters bronchial development, thus limiting the reliability of BLR for situs determination.

Our results corroborate Louise Calder’s postmortem study, which reported a smaller BLR value in situs solitus patients with RAA.^10^ Unlike Louise Calder’s study, our study on a larger cohort confirmed this finding with statistical significance. In heterotaxy, the bronchus on the same side of the aortic arch was longer than the opposite bronchus in the majority of cases, further supporting the influence of aortic arch position on bronchial length.

### Limitations

The retrospective design is associated with potential selection bias. The small number of patients with situs inversus and DAA limits statistical power and increases the risk of type II error. Consequently, the pairwise comparisons between the situs solitus and situs inversus groups must be interpreted with caution as the small sample size could affect the reliability and generalizability of the findings, potentially underestimating true differences between these groups.

## Conclusions

The BLR is influenced by the position of the aortic arch and is reduced when the arch is located on the atypical side for a given situs. Consequently, BLR has limited reliability for situs determination. The bronchopulmonary arterial relationship should be regarded as the primary criterion for determining thoracic situs.

## Data Availability

The data supporting the findings in this study are saved in the password-secured computer in the last author’s locked office per institutional research ethics guidelines.

## Ethics Statement

This research has adhered to the relevant institutional IRB ethical guidlines.

## Patient Consent

The authors confirm that patient consent is not applicable to this article. This is a retrospective case report using de-identified data; therefore, the IRB did not require consent from the patients.

## Funding Sources

The authors have no funding sources to declare.

## Disclosures

The authors have no conflicts of interest to disclose.
